# Consistency of quality attributes for the glycosylated monoclonal antibody Humira® (adalimumab)

**DOI:** 10.1080/19420862.2015.1073429

**Published:** 2015-07-31

**Authors:** Paul W Tebbey, Amy Varga, Michael Naill, Jerry Clewell, Jaap Venema

**Affiliations:** 1 AbbVie; Global Medical Affairs; Biologics Strategic Development; North Chicago, IL USA; 2 AbbVie; Biologics Development and Manufacturing Launch; AbbVie Bioresearch Center; Worcester, MA USA

**Keywords:** adalimumab, biosimilars, manufacturing, oligosaccharides, quality attributes

## Abstract

Humira® (adalimumab) is a recombinant human IgG1 monoclonal antibody (mAb) glycoprotein consisting of 1330 amino acids that is specific for human tumor necrosis factor (TNF). The biological activity and clinical profile of mAb therapeutics, including adalimumab, is influenced by their protein structure and glycosylation patterns, which can be affected by the expression system, cell culture conditions and purification process methodology. While clinical outcome cannot yet be attributed to many of the individual structural features that constitute a mAb, it is evident that detailed structural attribute analysis is necessary if structural contributions to function are to be comprehensively defined. Adalimumab product quality data generated from over a decade of manufacturing across multiple production sites and through a series of manufacturing scale changes are presented here. These data reveal a consistent and tightly controlled profile for the product.

## Abbreviations

mAbs: monoclonal antibodies
TNF: tumor necrosis factor
RA: rheumatoid arthritis
WCX-HPLC: weak cation exchange high performance liquid chromatography
NP-HPLC: normal phase high performance liquid chromatography
SD: standard deviation

## 

Monoclonal antibodies (mAbs) have transformed medicine by providing physicians and patients with selective therapeutic tools that were developed based on knowledge of disease pathogenesis. Adalimumab, a recombinant human IgG1 mAb that targets tumor necrosis factor (TNF), has been evaluated in more than 23,000 patients participating in ∼70 clinical trials.^1-4^ Marketed as Humira®, adalimumab was first approved in the US in 2002 for rheumatoid arthritis (RA), and since then it has been approved in numerous countries for RA, as well as other indications such as psoriatic arthritis, ankylosing spondylitis, Crohn's disease, plaque psoriasis, juvenile idiopathic arthritis, ulcerative colitis, non-radiographic axial spondyloarthropathy, as well as intestinal Behçet's disease in Japan.

The manufacturing process for a marketed biologic is typically modified several times during its lifecycle in order to increase process robustness, introduce new technology, introduce alternative raw material suppliers, or change production scale or site as a result of increased market demand. Product quality differences and shifts have been reported from such manufacturing changes with other biological products.^5,6^ The increasing demand for adalimumab, generated by virtue of expanding indications and patient use, has led to the need for expansion of manufacturing capacity via production scale increases and addition of new manufacturing sites. Although the biopharmaceutical industry has acknowledged that site or scale changes have led to shifts in product quality,^5^ the objectives for the production of adalimumab have been to assess the effect of proposed changes on product attributes and to maintain comparability with the quality history of the product. This approach has maintained the product quality “circle” that collectively represents the attributes of the molecule, the process, as well as the product quality and stability properties throughout the manufacturing history. Consequently, adalimumab's unique “fingerprint,” comprising a heterogeneous population of molecular species, has been well characterized and has not changed significantly throughout the clinical and commercial life of the product. As displayed in **Table 1**, subsequent to initial marketing authorization in the European Union in 2003, the adalimumab drug substance manufacturing process has been limited to increases in manufacturing scale, addition of new manufacturing sites as well as improvement of process controls, robustness and analytical methods to tighten specifications.^6,7^ The key element to maintaining a consistent product quality profile during commercial manufacture at 4 sites and 5 production scales for over a decade has been the global alignment of process controls, product quality testing and trending. This focus has resulted in the requisite maintenance of the structural integrity, purity and stability of adalimumab.^8^

**Table 1.** Manufacturing changes for Humira® (adalimumab) drug substance: EMA variations. Procedural details of steps taken to modify drug substance manufacture subsequent to initial authorization of Humira® (adalimumab) by EMA on 8th September, 2003. Abbreviations: EMA = European Medicines Agency; AS = active substance; FP = finished product; DS = drug substance, MCB = Master Cell Bank; WCB = Working Cell Bank; mfg. = manufacturing

Category	Variation	Scope	Description of change	Opinion date
Specs/controls	II/0003	Changes to test methods and/or specification for AS	Update test method and tighten specification	17 Dec 2003
Site/scale	II/0007	Changes to mfg. process for AS	6000L scale up	26 Feb 2004
Specs/controls	II/0011	Changes to mfg. process for AS	Qualify new WCB (same MCB)	29 Jul 2004
Process Robustness	II/0012	Changes to mfg. process for AS	Purification process alignment, 3000L/ 6000L	29 Jul 2004
Specs/controls	II/0013	Changes to shelf-life or storage conditions	Extend DS shelf-life to 36 months	18 Nov 2004
Specs/controls	II/0014	Changes to test methods and/or specification for AS	Update test method and tighten specification	18 Nov 2004
Specs/controls	II/0017	Changes to mfg. process for AS	Establish WCB qualification protocol	17 Feb 2005
Specs/controls	II/0018	Changes to mfg. process for AS	Updates to in-process controls	17 Feb 2005
Specs/controls	II/0020	Changes to mfg. process for AS Changes to test methods and/or specification for AS	Introduce new test method and establish specification; update in-process controls	21 Apr 2005
Process Robustness	II/0025	Changes to mfg. process for AS	Updates to in-process controls and enhancements to make the process more robust	26 Jan 2006
Specs/controls	II/0031	Changes to test methods and/or specification for AS	Introduce new test method and revise specification	27 Jul 2006
Site/scale	II/0036	Changes to mfg. process for AS	Addition of DS manufacturing site and scale (12000L)	22 Mar 2007
Specs/controls	II/0041	Changes to test methods and/or specification for AS	Update method descriptions	17 Jul 2007
Specs/controls	II/0054	Changes to test methods and/or specification for AS	Update test method and tighten specification	22 Jul 2008
Specs/controls	II/0065	Changes to AS shelf life	Extend DS shelf life to 60 months	6 Jul 2009
Site/scale	II/0070	Changes to mfg. process for AS	Addition of new DS manufacturing site and scale (10000L) and enhancements to make the process more robust	18 Mar 2010
Site/scale	II/0095G	Changes to mfg. process for AS	Addition of new DS manufacturing site and scale (20000L)	21 Feb 2013
Specs/controls	II/0099G	Changes to specification limits for AS and FP	Tighten specification	13 Dec 2012
Raw materials/ commodities	II/0116	Changes to mfg. process for AS	Qualification of new raw material source	27 Jun 2013
Raw materials/ commodities	II/0119	Changes in manufacturer of starting materials/reagents for AS	Qualification of new raw material source	23 Jul 2013
Process Robustness	II/0121	Changes to mfg. process for AS	Alignment of purification process across sites	19 Sept 2013

The complex structures of biologic products are not easily characterized.^9^ Therapeutic proteins possess structural features that if modified, even slightly, may affect clinical performance. Consequently, quantification of the breadth of physicochemical and biologically based attributes is needed in order to maintain product quality. To accomplish this in large-scale manufacturing processes, it is important to understand which molecular characteristics are most sensitive to various production process factors. Two properties most often used to assess consistency in the production process and the effect of process change are molecular charge and glycosylation.^5,10-15^ Microheterogeneity, which is typical of mAbs, occurs as the protein is being expressed by the cells in culture and is related to the type of cell used in production and the process culture conditions, e.g., media composition, time, aeration, pH and temperature.^16^ Charge variants can include molecular species that exhibit differences in size or charge as a consequence of oxidation, deamidation, isomerization, glycation or C-terminal lysines driven by cellular enzymatic processes or spontaneous degradation.^17^ Variation in molecular charge may influence antibody binding affinity, and thus affect potency, aggregate formation, serum half-life, or stability upon storage of the drug product.^18^ Specific glycosylation patterns (i.e., the composition of attached sugar moieties) of antibodies can also be used as an indicator of a consistent manufacturing process or to identify the effect that changes to the manufacturing process have on the molecule.^19,20^ Characterized by the degree of galactose sugar molecules, one of the major glycoforms of mAbs, including adalimumab, is the agalactosyl fucosylated biantennary oligosaccharide (G0F) species that contains fucose but no terminal galactosylation.^21^ Evidence that glycosylation patterns affect protein function is accumulating, and differential glycosylation has been shown to influence serum clearance in human studies of therapeutic antibodies with oligomannose sugars,^21-23^ antibody-dependent cell-mediated cytotoxicity or complement-dependent cytotoxicity, ^18,21,24^ and serum half-life due to differences in binding to the Fc receptors.^10,12,13,15^

**Figure 1.**
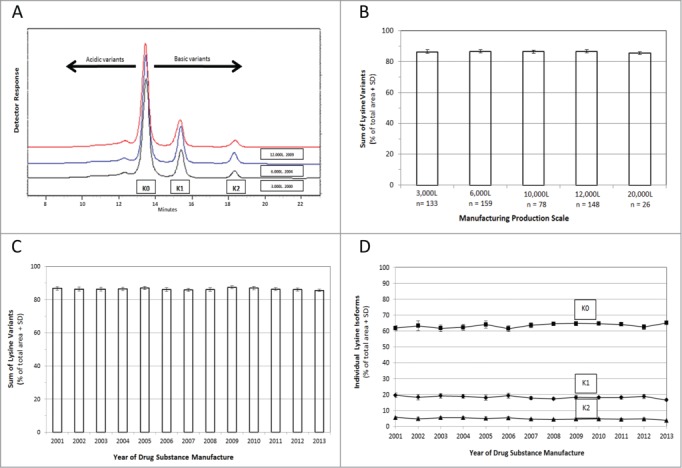
Lysine Profiles of Humira®. Chromatograms of representative batches are displayed in **A**; 3,000L (Black; 2000), 6,000L (Blue; 2004), 12,000L (Red; 2009). WCX-HPLC was performed on batches of Humira that derived from scale-up production (3,000 to 20,000 liters, **B**) and through each year 2001 to 2013 (**C & D**). The chromatograms illustrate the relative retention time and relative peak areas. The relative amount of the 3 C-terminal lysine isoforms (K0, K1, K2) was calculated from the chromatograms as a percent of total area. The mean sum of lysines of multiple batches per data point is presented with standard deviation (n = 544 batches for **B** and 525 total batches included for **C and D**). The number of drug substance batches evaluated per data point is displayed in **B**. For each year 2001 to 2013 (**C and D**), the number of batches included in each data point is 13, 38, 50, 44, 54, 40, 37, 34, 24, 34, 57, 52, 48, respectively. The mean of individual lysine species (K0 [square], K1 [diamond] & K2 [triangle]) is presented with standard deviation (**D**).

In the case of adalimumab, manufacturing changes have included scale increases and site transfers, specification changes, or process control improvements across manufacturing sites.^6,7^ To assess the charge species for adalimumab drug substance production, weak cation exchange high performance liquid chromatography (WCX-HPLC) was used to separate isoforms of adalimumab molecules containing acidic species, C-terminal lysine variants (zero lysine, K0; one or 2 lysines, K1 or K2, respectively) or other minor species based upon differences in the density of surface charge. The focus on antibody drug substance facilitates assessment of the fundamental properties of the mAb, which are established through the generation and use of a product-specific cell line and master cell bank, the cell culture conditions employed and the purification process. Although C-terminal lysines are generally cleaved in vivo, alterations in the proportion of C-terminal lysine species represent a signature of process consistency or indicate unexpected changes in overall molecular charge.^11^ Representative chromatograms of adalimumab reference standards produced at different sites, scales and time periods are displayed in **Figure 1A**. The data illustrating the 3 lysine variants (K0, K1 and K2), as well as the small acidic peaks in the reference standard samples, show that there is minimal variability in the peaks of the chromatograms, and no new peaks are seen as the process was scaled up from the 3,000 L bioreactor scale to the 12,000 L bioreactor scale; similar results were obtained at the 20,000L bioreactor scale (**Fig. S1**). Also shown is a comprehensive survey of the charged species across the manufacturing history from clinical batches prior to the initial registration of adalimumab in 2002 through production in 2013. The sum of the lysine variants are illustrated in **Figure 1B** for the 5 production bioreactor scales, and **Figure 1C** for production from 2001 through 2013. **Figure 1D** provides further evidence of consistency within the individual lysine species (K0, K1 and K2) across the same production timeframe. **Figure S2** displays the sum of lysines for individual batches through time and by bioreactor scale. Collectively, these data demonstrate that the charge microheterogeneity of adalimumab drug substance, as measured by the proportion of lysine variants, has been consistently and tightly maintained.

**Figure 2.**
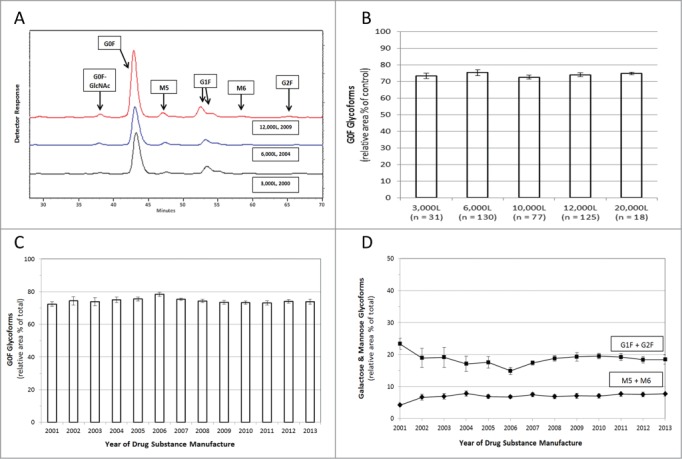
Glycan Mapping of Humira®. Assessment of oligosaccharides was performed using NP-HPLC to separate fluorescent-labeled glycan species. (**A**) Representative chromatograms of Humira® reference standards from different scale / years displaying glycan elution patterns (3,000L (Black; 2000), 6,000L (Blue; 2004), 12,000L (Red; 2009). The chromatograms illustrate the relative retention time and relative peak areas. The relative amount of each glycan species was calculated from the chromatograms as a percent of total area. (**B**) Agalactosyl fucosylated biantennary oligosaccharides (G0F) are displayed by scale-up production (3,000 to 20,000 liters; mean ± standard deviation, total n = 381). (**C**) Agalactosyl fucosylated biantennary oligosaccharides (G0F) were analyzed through time (mean plus standard deviation). The number of drug substance batches (total n = 381) evaluated per data point for each year 2001 to 2013 is 4, 6, 8, 15, 45, 18, 29, 34, 30, 43, 55, 46, 48, respectively (**C and D**). **(D**) Galactose-containing fucosylated biantennary oligosaccharides (G1F + G2F) are plotted (squares) with detectable oligomannose species (M5 + M6) (diamonds) with each data point displaying 1 SD of the mean.

**Figure 3.**
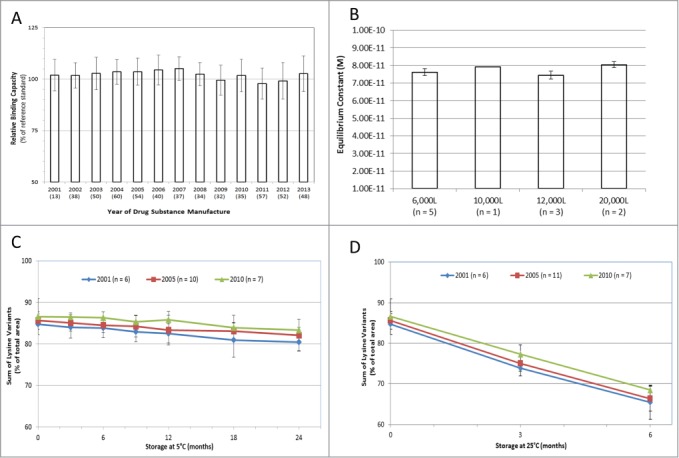
Binding Affinity and Stability Profiles of Humira®. The binding capacity of Humira® drug substance to recombinant human TNF was assessed by anti-TNF ELISA (**A**) and by surface plasmon resonance (**B**). For anti-TNF ELISA, test batches are represented from each year of manufacture from 2001 to 2013 (n = number of batches per data point). Results are presented as the percent binding capacity of the test sample relative to that of the reference standard (black bars) plus standard deviation. Intrinsic binding affinities of adalimumab were determined by surface plasmon resonance via measurement of the rates at which soluble TNF bound to and dissociated from single antigen-binding sites of surface-bound adalimumab. The graph displays the molar equilibrium constant for batches of adalimumab by manufacturing scale (total n = 11) (**B**). Sum of lysine variants of adalimumab drug product stored at 5°C for up to 24 months as measured by WCX-HPLC (**C**). Samples derive from 2001 (diamond, n = 6), 2005 (square, n = 10) and 2010 (triangle, n = 7). The mean sum of lysines is presented with standard deviation. Sum of lysine variants of Humira® drug product stored at 25°C for up to 6 months as measured by WCX-HPLC (**D**). Samples (Mean ± SD) derive from 2001 (diamond, n = 6), 2005 (square, n = 11) and 2010 (triangle, n = 7).

The identity and quantity of the oligosaccharides on the conserved N-linked glycosylation site of adalimumab drug substance was evaluated by normal phase high performance liquid chromatography (NP-HPLC) on manufacturing batches from 2001 to 2013. Representative chromatograms from different manufacturing scales are displayed in **Figure 2A** and **Figure S3**. The relative quantity of the agalactosyl fucosylated biantennary oligosaccharides in adalimumab, including forms lacking a terminal N-acetyl glucosamine (i.e., both G0F and G0F-GlcNAc) has remained remarkably consistent through production scale-up (**Fig. 2B**) and time (**Fig. 2C**), as have the proportions of galactose-containing fucosylated biantennary oligosaccharides (G1F + G2F) and detectable high mannose glycoforms (M5 + M6) (**Fig. 2D**). **Figure S4** displays the G0F oligosaccharides in adalimumab for individual manufacturing batches through time and by bioreactor scale. The presence of high mannose glycans has been shown to stimulate enhanced clearance of mAbs, and thus should be considered an important product quality attribute when determining comparability.^25^ In addition to an analysis of the structure of adalimumab, the potency of the antibody for ligand binding has also been assessed. Adalimumab binds with high affinity to soluble TNF as measured by an anti-TNF ELISA in which recombinant human TNF was used to capture the serially-diluted drug substance samples compared with a reference standard to determine percent binding capacity (**Fig. 3A**). During more than a decade of production, the TNF ligand binding results consistently ranged within 7 percentage points of the established reference standard. The intrinsic binding affinity of adalimumab for soluble TNF, as measured by surface plasmon resonance, displayed a mean dissociation constant (K_D_) of 7.66 × 10^−11^ M, and was consistent for production batches ranging from 6,000 to 20,000 L in scale, as well as for batches that were manufactured from 2003 to 2011 (**Fig. 3B**). The consistency in ligand binding and affinity for soluble TNF translated into functional potency of the antibody to prevent TNF-induced cell cytotoxicity (**Fig. S5**).

Of importance is whether the consistency of the adalimumab quality attribute profile is maintained at the level of both the drug substance as well as drug product. A very useful indicator is the assessment of drug product stability under the conditions of normal storage (5°C) and under stressed conditions (25°C). In the data presented here, the change in the sum of lysine charge variants over the stability period was used to illustrate the consistency of the drug product profile. Changes in charge variants can occur as the mAb is produced by the cells (e.g., post-translational modification of the protein), throughout purification, or during storage in the final container (e.g., a pre-filled glass vial or syringe). The environment that the mAb is exposed to (i.e., formulation buffers, trace impurities, time and temperature of storage) can affect the kinetics of change in charge variants. Thus, a consistent stability profile, i.e., the rate of change in the level of the charge variants, is a sensitive indicator of stable composition. The sum of the lysines profile containing K0, K1 and K2 isoforms was used as a proxy to measure the charge variability profile (**Fig. 3C** and **3D**). Drug product samples (n = 23) were sourced from lots manufactured in 2001, 2005 and 2010 and were stored at 5°C for up to 24 months (**Fig. 3C**). The data demonstrate that the sum of lysine stability profile was consistent for all drug product samples over the 24-month period. Under stressed storage conditions of 25°C, any changes that may occur upon storage will be accelerated, and thus will provide a clear indicator of potential differences in the stability profile. **Figure 3D** illustrates that the accelerated stability profile of the drug product samples is consistent throughout the 10-year manufacturing window of analysis.

Little to no data has been published for other marketed biologics, and the data that is available indicates a relatively broad window for acceptable variations.^5^ In contrast, we present a series of quality attributes for adalimumab that display a consistent and tightly controlled manufacturing profile over more than a decade of production at 5 manufacturing scales and 4 manufacturing sites, regardless of multiple process amendments. This comprehensive, orthogonal analysis illustrates that the microheterogeneity displayed by adalimumab is both temporally consistent and remains within a narrow range of variability in physicochemical and functional quality attributes. From 2002 to 2013, there were a total of 13 batches of adalimumab drug substance that were not released to the market due to specification failures of process-related impurities. The quality attribute data presented in this manuscript represent the remaining 98% of the batches that were produced and released to the market. Such a batch failure rate is sufficiently low to have no impact on the analysis of attribute consistency presented herein.

In addition to furthering the understanding of individual structural attributes that constitute a mAb, these data highlight the value of profound comprehension of structural components of mAb reference products, as well as their biosimilars, since they relate to in vivo mechanism of action and clinical performance. Typically, regulatory guidance documents on biosimilar products recognize the criticality of establishing the analytical window of similarity, but quantitative definitions on the level of similarity to the attribute-based range of the reference product are largely lacking.^6,9,14^ The data shown here helps to define the important role of analytical and functional characterization in controlling process and product limits for antibody products over time.

## Materials

Drug substance batches of Humira® (adalimumab) manufactured from 2000 to 2013 were utilized for this analysis (544 total batches). The drug substance batches derived from multiple scales (3,000; 6,000; 10,000; 12,000 and 20,000 L) and came from manufacturing facilitates in the US, Puerto Rico, Spain and Singapore. No differences in attribute analysis were observed between manufacturing sites. The relevant number of batches produced per year is displayed in the appropriate figure legends.

Cation Exchange HPLC: Weak cation exchange HPLC (WCX) was used to separate variants of the Humira® molecule based upon differences in net surface charge density. Drug substance samples and reference standard were analyzed using a Dionex ProPac™ WCX-10 analytical column (Thermo Scientific, Sunnyvale, CA) containing ethylvinylbenzene-divinylbenzene polymer incorporating a protective guard column (ProPac WCX-10G). Equal volumes of both reference standard and sample(s) were injected onto the column and components were eluted using a linear gradient from 6% to 16% solvent B in 34 min (Solvent B: 10 mM sodium phosphate dibasic, 500 mM sodium chloride, pH 5.5; Solvent A: 10 mM sodium phosphate, pH 7.5). Protein elution was monitored at a wavelength of 280 nm. A representative WCX chromatogram of Humira® reference standards is displayed in **Figure 1A**.

Supplemental_Material.docx

## Analysis of Oligosaccharides

The identity and quantification of the oligosaccharides on the conserved N-linked glycosylation site of Humira® was performed by N-glycanase cleavage and normal phase separation (NP-HPLC) with fluorescent detection (Signal™ 2-Aminobenzamide labeling kit, ProZyme, San Leandro, CA). The labeled glycans were separated by NP-HPLC (ProZyme GlycoSep™ column) using acetonitrile (mobile phase A) and 50 mM ammonium formate, pH 4.4 (mobile phase B). Quantitation was based on the relative area percent of detected sugars. The percentage relative area of agalactosyl fucosylated biantennary oligosaccharides (NGA2F + [NGA2F-GlcNAc]), galactose-containing fucosylated biantennary oligosaccharides (NA1F + NA2F), oligomannoses (M5 + M6) and the proportion of NA1F form as a percent of total galactose-containing fucosylated biantennary oligosaccharides in the sample(s) are reported. Representative chromatograms of Humira® reference standard glycan patterns are shown in **Figure 2A**.

Anti-TNF ELISA: Anti-TNF ELISA was used to determine the relative binding capacity of Humira® in solution. Recombinant human TNF was used to capture both drug substance samples and Humira® reference standard. Goat anti-human IgG conjugated to horseradish peroxidase (HRP) was then added to detect bound Humira® captured by the coating protein. Subsequent to washing to remove unbound conjugate, the chromogenic substrate, tetramethylbenzidine was added to the plate. Reaction quenching was achieved via acidification with sulfuric acid prior to absorbance readings at 450 nm. A four-parameter fit model was used to determine BC50 values [i.e., equivalent to the log concentration of Humira® at which 50% of antigen is bound to the antibody]. The ratio of the resulting BC50 values for the reference standard and drug substance sample curves yielded a decimal fraction that is converted to a percent binding capacity of the sample relative to that of the reference standard.

### Affinity of binding of soluble TNF to immobilized adalimumab

Surface plasmon resonance-based measurements were obtained with a BIAcore® 3000 instrument (GE healthcare), essentially as previously described using a dextran biosensor matrix on a gold surface. Goat anti-human IgG (Fcγ) fragment-specific polyclonal antibody was immobilized on the biosensor chip and adalimumab was injected over the reaction matrix in a continuous flow system. Triplicate runs were performed for all lots. The net difference in response units (RU) at baseline and after antibody injection represented the mass of bound adalimumab. Levels of TNF bound to adalimumab were recorded as a function of time in sensorgram plots. For kinetic analysis, rate equations derived from the 1:1 Langmuir binding model were fitted simultaneously to association and dissociation phases of all of the runs (using global fit analysis) with the use of T100 evaluation software, version 2.0.1. Kinetic rate constants for association rate constant (Kon, M-1s-1) and dissociation rate constant (Koff, s-1) were determined under a continuous flow rate of 50 μL/min. Rate constants were derived by making kinetic binding measurements at 7 different antigen concentrations ranging from 0.78-50 nM. The equilibrium dissociation rate constant (K_D_, M) of the reaction between adalimumab and recombinant human TNF was calculated from the kinetic rate constants by the formula: K_D_ = koff /kon.

## L929 Potency Assays

The neutralizing potency of adalimumab was tested in the mouse fibroblastic cell line L929 (American Type Culture Collection, Rockville MD) using human TNF. Various concentrations of adalimumab were mixed with a constant amount of TNFα before being added to the L929 cells. Cell viability was measured using the tetrazolium dye, MTT. The potency of adalimumab was determined by comparing its neutralizing effect on the cytotoxicity of human TNF on L929 cells relative to an adalimumab reference standard. The IC_50_ was determined for both adalimumab reference standard and test samples to calculate the % potency (i.e., the ratio IC_50_ (reference standard) divided by IC_50_ (test sample).
