# Supplementary material for: Consistency of quality attributes for the glycosylated monoclonal antibody Humira® (adalimumab)
Source: MAbs. 2015 Jul 31;7(5):805–11. doi: 10.1080/19420862.2015.1073429 (PMC4622832; doi:10.1080/19420862.2015.1073429)
Supplement: Supplemental_Material.docx [file kmab-07-05-1073429-s001.docx]

**Supplementary Materials: Consistency of quality attributes for the glycosylated monoclonal antibody Humira® (adalimumab) by Paul W. Tebbey, Amy Varga, Michael Naill, Jerry Clewell & Jaap Venema**

**Supplementary Figure 1.** CEX-HPLC chromatograms displaying the charge variant profiles of adalimumab batches from different manufacturing scales and years.


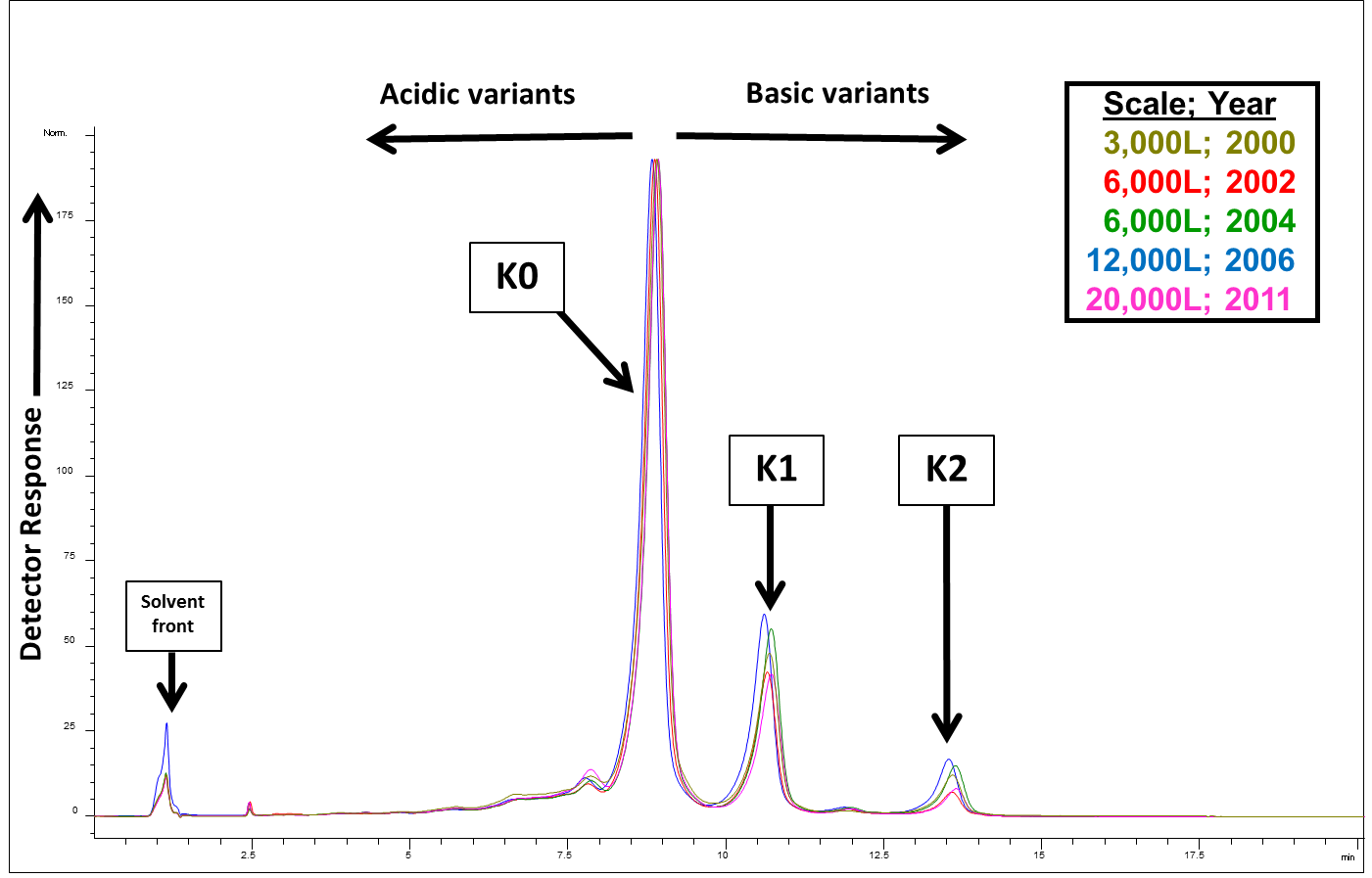


**Supplementary Figure 2.** CEX-HPLC scatter plots of sum of lysine charge variants displaying individual batches of adalimumab by scale and through time.

**Supplementary Figure 3.** NP-HPLC chromatograms displaying the oligosaccharide profiles of adalimumab batches from different manufacturing scales and years.


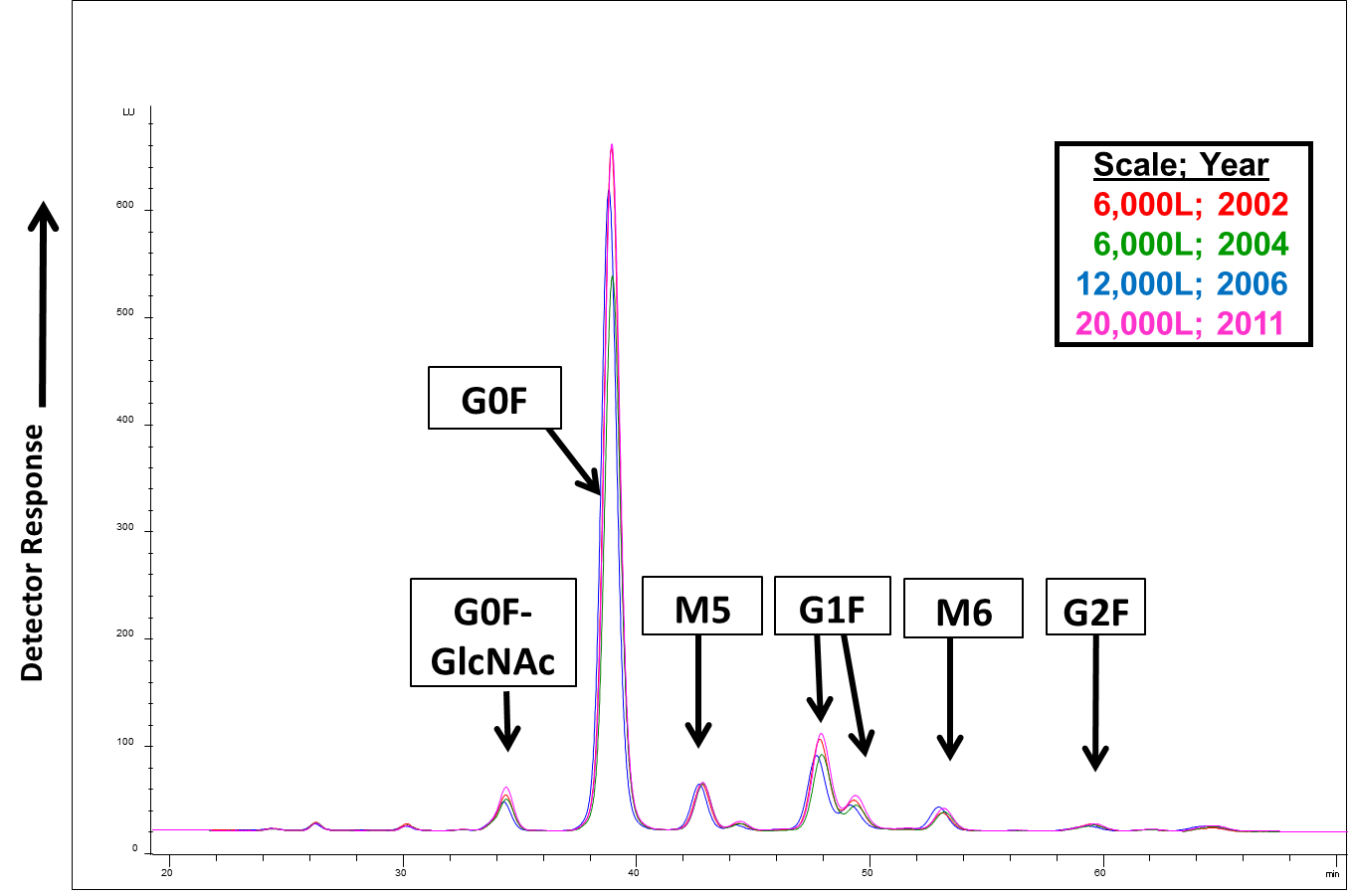


**Supplementary Figure 4.** NP-HPLC scatter plots of total agalactosyl fucosylated biantennary oligosaccharides (G0F) displaying individual batches of adalimumab by scale and through time.

**Supplementary Figure 5.** Potency of adalimumab inhibition of TNF-induced L929 cell cytotoxicity
